# A trial of nurturing care among children who are HIV‐exposed and uninfected in eSwatini

**DOI:** 10.1002/jia2.26158

**Published:** 2023-11-01

**Authors:** Andrea Ruff, Xolisile Dlamini, Bareng AS Nonyane, Nicole Simmons, Duncan Kochelani, Fiona Burtt, Fakazi Mlotshwa, Ncamsile Gama, Esca Scheepers, Kathrin Schmitz, Lethokuhle Simelane, Lynn M. Van Lith, Maureen M. Black

**Affiliations:** ^1^ Johns Hopkins Bloomberg School of Public Health Johns Hopkins University Baltimore Maryland USA; ^2^ Ministry of Health Kingdom of eSwatini South Africa; ^3^ Johns Hopkins Center for Communication Programs Baltimore Maryland USA; ^4^ mothers2mothers Cape Town South Africa; ^5^ University of Maryland School of Medicine Baltimore Maryland USA; ^6^ RTI International Research Triangle Park North Carolina USA

**Keywords:** CHEU, child development, HIV prevention, intervention, mothers to mothers, nurturing care

## Abstract

**Introduction:**

Children who are HIV‐exposed and uninfected (CHEU) are a growing population at potential risk of poor neurocognitive development. We tested a nurturing care intervention on children's neurocognitive development and maternal depressive symptoms (primary) with mediation through caregiving activities (secondary).

**Methods:**

This study was conducted among six intervention and nine comparison antenatal‐care/prevention of vertical transmission (ANC/PVT) HIV clinics in eSwatini. We enrolled pregnant women and measured infant development at 9 and 18 months. mothers2mothers (m2m) designed and implemented the clinic‐home‐community‐based intervention. We measured infants’ neurodevelopment, maternal depressive symptoms and caregiving activities with the Mullen Scales of Early Learning (MSEL), Edinburgh Postnatal Depression Scale, HOME Inventory and Family Care Indicators. We fitted linear mixed effects regression models with clinic random effects to compare intervention versus comparison arms, and generalised structural equation models to evaluate mediation, adjusting for confounders.

**Results:**

Mother‐infant pairs (*n* = 429) participated between January 2016 through May 2018. Socio‐demographic characteristics were balanced between arms except for higher rates of peri‐urban versus rural residence and single versus married mothers in the comparison group. The 18 month retention was 82% (180/220) intervention, 79% (166/209) comparison arm, with 25 infant deaths. Intervention MSEL scores were significantly, and modestly, higher in receptive language (55.7 [95% CI 54.6, 56.9] vs. 53.7 [95% CI 52.6, 54.8]), expressive language (42.5 [95% CI 41.6, 39.8] vs. 40.8 [95% CI 39.8, 41.7]) and composite MSEL (85.4 [95% CI 83.7, 84.5] vs. 82.7 [95% CI 81.0, 84.5]), with no difference in maternal depressive symptoms or in observations of mother‐child interactions. Intervention book‐sharing scores were higher (0.63 vs. 0.41) and mediated the effect on MSEL scores (indirect effect, *p*‐values ≤ 0.024). The direct effects on visual reception and expressive language scores were significantly higher in the intervention compared to the comparison arm (coefficients 1.93 [95% CI 0.26, 3.60] and 1.66 [95% CI 0.51, 2.79, respectively]).

**Conclusions:**

Nurturing care interventions can be integrated into ANC/PVT clinic‐home‐community programmes. The intervention, mediated through interactive caregiving activities, increased language development scores among CHEU. Partnering with a local team, m2m, to design and implement a culturally relevant intervention illustrates the ability to impact parent‐child play and learning activities that are associated with children's neurodevelopment.

## 1 INTRODUCTION

Paediatric HIV acquisitions have declined significantly, largely due to the expansion of antenatal‐care/prevention of vertical transmission (ANC/PVT) programmes [1]. The population of children who are HIV‐exposed and uninfected (CHEU) has increased substantially, with risks of morbidity and mortality, preterm birth, growth faltering and potential effects on neurodevelopment [2]. Several studies found no differences in neurodevelopmental scores between uninfected children living with and without HIV exposure [3, 4, 5, 6], but others [7, 8, 9], including two meta‐analyses [10, 11], found lower neurodevelopmental scores among CHEU, compared to children who are HIV unexposed. Variability in samples and environmental exposures associated with HIV, such as stress and depression, limited coping strategies and economic resources, stigma, violence, and drugs and alcohol, may explain the variable results [12].

Early neurodevelopment lays the foundation for academic and economic capabilities [13]. Nurturing care [14] supports children's development and promotes supportive caregiving along the life‐course from conception through childhood and into adulthood [15]. During infancy, caregiving activities, including book‐sharing and story‐telling, have been positively associated with neurodevelopment [16, 17, 18]. Early child development (ECD) programmes have demonstrated significant improvements in children's neurodevelopment [19, 20], including for children with HIV [21, 22]. Nurturing care interventions have been recommended for all children [14] and may be effective among CHEU.

eSwatini's introduction of life‐long antiretroviral therapy (ART) for pregnant women provided an opportunity to test the effectiveness of a nurturing care intervention among mothers with HIV and their exposed children within a national PVT programme. We tested two hypotheses: that a nurturing care‐based intervention would improve children's neurodevelopment and mothers’ mental health (primary) and that caregiving activities would mediate the effects on neurodevelopment (secondary).

## 2 METHODS

**Figure 1:**
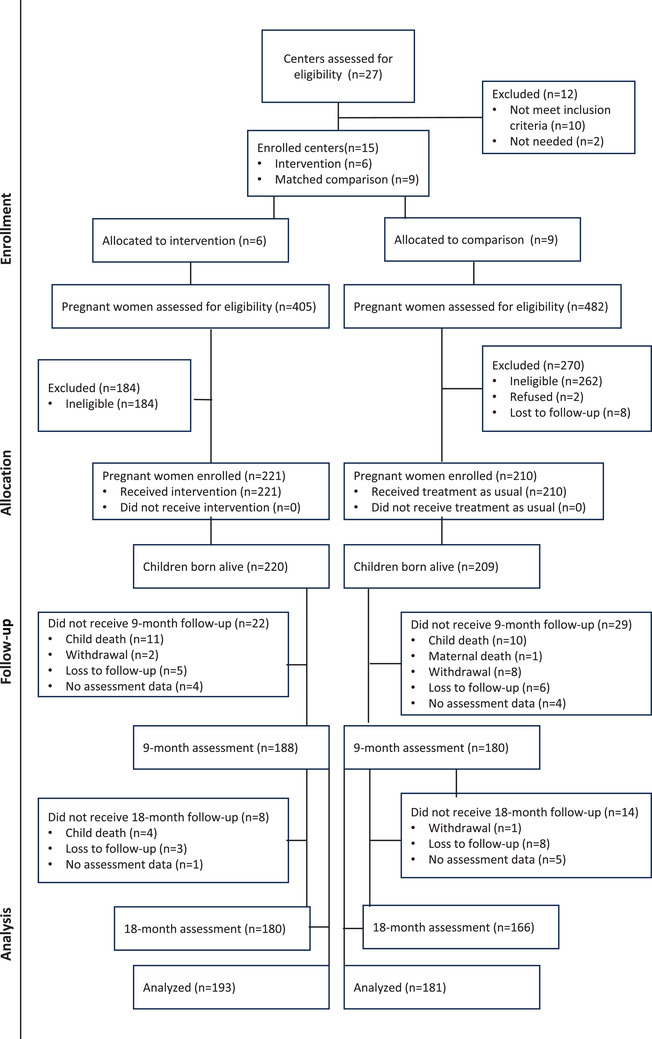
Consort diagram.

We implemented a design using six mothers2mothers (m2m)‐supported clinics (intervention) and nine clinics supported by PEPFAR partners (comparison), matched on providing PVT under the national AIDS programme, lifetime ART, comparable number of women HIV positive, DNA PCR testing for children and location within 2.5 hours of the capital, Mbabane. Exclusion criteria were high rates of client mobility and implementation of nurturing care interventions (Figure 1).

### 2.1 Intervention

We collaborated with m2m, an African nonprofit organisation supporting women, children and their families which is integrated into eSwatini national programmes [23]. m2m designed and implemented the intervention by translating nurturing care principles into culturally appropriate activities (Table S1). They hired women living with HIV as Mentor Mothers [24] and trained them in maternal care and ECD, including sensitivity to women living with HIV, using a home‐based intervention, and behaviour change strategies, a reference manual, a flipbook with key messages, and culturally and age‐appropriate information for families.

The nurturing care intervention included home visits (biweekly through infant age 12 months, monthly through 24 months), community parenting groups and individual counselling during clinic visits. Mentor Mothers were trained to maintain friendly, mutually respectful and positive relationships with participating mothers and families. They discussed growth stages, provided picture books and coached caregivers on interactive play, story‐telling, and book‐sharing and recorded contacts in family folders and visit checklists. The intervention began prenatally and continued through the infant age 2 years. Comparison clinics received usual care.

### 2.2 Sample size and study design

To estimate sample size, we used the age‐normed standardisation sample of the Mullen Scales of Early Learning (MSEL) (mean 100, standard deviation 15), with a difference of 0.5 standard deviations (7.5 points). Participants were selected by site (cluster). We used the clustersampsi procedure in Stata version 12 [25], initially using six clusters per arm. Based on a similar ECD study [26], we set the intra‐cluster correlation at 0.04 and allowed for a coefficient of variation among cluster participants of 0.31, resulting in an average of 27 participants per cluster to achieve 80% power and 7.5 points between‐arm difference, with 0.05 Type 1 error. Repeating the estimate with nine clusters per arm, an average cluster size of 15, and a coefficient of variation of 0.29, yielded an intra‐cluster correlation up to 0.06 with 80% power to detect 7.5 points between arms with 0.05 Type 1 error. We over‐enrolled to allow for 30% loss‐to‐follow‐up.

### 2.3 Procedures

Participants were recruited from ANC clinics. Inclusion criteria were third trimester of pregnancy, confirmed HIV positive, intention to remain in the clinic catchment area for 18 months and ability to consent. Mentor Mothers and nurses informed clinic participants about the study; research staff verified eligibility and obtained informed consent. Ethical approval was granted by Institutional Review Board at the Johns Hopkins Bloomberg School of Public Health and the Scientific and Ethics Committee of the Ministry of Health in eSwatini; written consent was obtained from all participants. Between January and August 2016, we enrolled pregnant women and followed them through the child age 18 months. We collected data using tablets with Magpi software.

The evaluation team included site coordinators who conducted routine follow‐up visits, and ECD assessors, who were unaware of intervention status. At enrolment, site coordinators collected demographic and contact information, abstracted data from patient‐held HIV and maternal health cards and administered baseline assessments.

Two weeks after delivery and at child ages 3, 6, 9 and 18 months, site coordinators conducted clinic‐based follow‐up interviews with mothers. At 12–15 months, site coordinators conducted home‐visit observations. At 9 and 18 months, ECD assessors measured children's neurodevelopment and growth, and maternal depression and caregiving practices. To ensure that ECD assessors were unaware of the study arm, children and caregivers were transported to one of three centralised assessment stations that served both intervention and comparison arms; ECD assessors had no other contact with participants or children during the study.

### 2.4 Measures

We selected measures that captured children's neurodevelopment and represented contextual variables from the nurturing care framework that support neurodevelopment, including maternal depression, caregiving behaviours and the home environment. We prioritised measures that had been standardised and validated with mothers and young children globally, preferably in Africa.

Items from all measures were culturally adapted for eSwatini, translated into siSwati and back‐translated. We conducted pilot testing on non‐study mothers and children from well‐child visits, with modification to materials and procedures as necessary to ensure cultural appropriateness while retaining rigour.

#### 2.4.1 Neurodevelopment

We evaluated neurodevelopment using the MSEL [27], an individually administered assessment with excellent psychometric properties, used with CHEU in Uganda and Malawi [4]. It measures fine motor, gross motor, visual reception, receptive language and expressive language domains, and composite MSEL general cognitive ability. Raw scores were converted to age‐adjusted standard scores, based on published norms (mean = 100, standard deviation [SD] = 15). Comparisons between arms were interpreted from a population perspective, based on SD differences in the distribution. ECD assessors were trained by a licensed child development professional, supplemented by MSEL observations conducted among CHEU in a companion project in Zimbabwe. Inter‐rater reliability (Kappa) among 40 children scored by both ECD assessors at each time point ranged from 0.980 to 0.996.

#### 2.4.2 Growth

Site coordinators measured length (cm), weight (kg) and mid‐upper arm circumference (cm) in triplicate using a digital scale and stadiometer. Measurements were converted into WHO growth standards z‐scores: weight‐for‐age, length‐for‐age, weight‐for‐length and mid‐upper‐arm circumference‐for‐age [28]. Underweight, wasting and stunting were defined as < −2 weight‐for‐age, weight‐for‐length and length‐for‐age z‐scores, respectively.

#### 2.4.3 HIV testing

Site coordinators assessed infant HIV testing abstracting data from participant‐held maternal health/ANC and HIV cards and well childcare and child HIV cards, postnatal care and child welfare registers at the clinics, and caregiver‐report. Over 90% of children had a DNA PCR test done within 60 days of age. Compliance at 9 months was: intervention 65% (131/201) and comparison 57% (110/192). At 18 months, overall compliance was 43% (167/386) with little difference between study arms.

#### 2.4.4 Maternal mental health

Maternal depressive symptoms were measured using the Edinburgh Postnatal Depression Scale (EPDS) [29], a 10‐item measure of depressive symptoms, validated in South Africa [30]. The 4‐point response choices (0–3) were summed, with scores of 0–30. High scores indicate increased symptom severity. We defined scores ≥13 as depression based on findings of 76.0% sensitivity, 81.8% specificity and 57.6% positive predictive value among South African women [31].

#### 2.4.5 Household characteristics and caregiving practices

Household information was obtained during the baseline interview. The Family Care Indicator scale (FCI) [32, 33] was used to measure maternal caregiving activities: in the last 3 days, an adult engaged in six caregiving activities (reading/looking/sharing picture books, telling stories, singing songs, taking child out from house, playing with toys and naming/counting/drawing). Scores were yes (1) or no (0).

The Infant‐Toddler version of the Home Observation for the Measurement of the Environment (HOME) inventory was used to assess mother‐child interactions [34]. After pilot testing, we retained 16 Yes/No items. During the 12‐ to 15‐month home visit, site coordinators spent 40 minutes observing mother‐child interactions. Scores were summed; high scores indicated more responsive mother‐child interactions. Inter‐rater reliability (Kappa) exceeded 0.90.

#### 2.4.6 Contamination assessment

Participants were asked about their contact with m2m and exposure to ECD information.

### 2.5 Statistical analyses

We fitted linear mixed models for neurodevelopment scores at 9 and 18 months separately and across both time points, with random effects for clinic and child‐within‐clinic, and we evaluated time by arm interactions. We evaluated potential confounding of demographic variables on composite MSEL by fitting separate regressions, including study arm and potential confounders. We defined confounders as changing the mean study arm effects by >10% and changing the inference (confidence interval and *p*‐value).

We conducted a contamination sensitivity analysis by excluding comparison arm mothers who reported m2m/ECD contact and re‐running the linear mixed models for composite MSEL.

For maternal depression, we present the numbers and percentages of mothers with EPDS scores ≥13 at baseline (enrolment), 9 and 18 months. We used log‐binomial generalised estimating equations to test whether changes in depression prevalence differed by study arm over time, and linear mixed models to evaluate whether baseline maternal depression was associated with composite MSEL.

To analyse the quality of mother‐child interactions, we report the median (interquartile range) of HOME Inventory items observed. To analyse the FCIs, we report percentages (95% confidence intervals) of mother‐endorsed caregiving activities (0–6).

We fitted a generalised structural equation model (GSEM) in STATA to evaluate mediation by caregiving activities on the relation between study arm and 18‐month MSEL scores, adjusting for marital status and setting (rural vs. peri‐urban). For parameter estimation, we used STATA's default mean‐variance adaptive Gauss‐Hermite quadrature estimation and 1001 iterations. The GSEM model included two paths, (1) an indirect path of a generalised linear model (glm) with a logit link for associating caregiving activity and study arm (binary outcome and mediator), and a Gaussian model within an identity link for MSEL score regressed on caregiving activity and (2) a direct path linking MSEL score regressed on study arm. Robust variance estimation accounted for site clustering. Total, direct and indirect coefficients were extracted using STATA's nlcom (non‐linear combinations of estimators).

## 3 RESULTS

We enrolled 431 pregnant women (221 intervention and 210 comparison) (Figure 1) with 429 children born alive (220 intervention and 209 comparison). Retention at the 18‐month evaluation was 82% (180/220) intervention and 79% (166/209) comparison. Two participating mothers died, one per arm and 26 children died (16 intervention, 10 comparison). Two children (0.5%) had positive HIV tests, one per arm, and were retained. Most mothers accompanied their children to the assessments (9 months, 95.9% and 18 months, 87.5%). Attempts were made to contact non‐attending mothers by telephone to complete the assessments. Retention in well childcare did not differ by arm: 161/220 (73.2%) intervention and 140/209 (67%) comparison.

Arms were balanced across number of children, maternal education, employment, timing of maternal ART treatment (54.5% prior to pregnancy), socio‐economic status and utilities, including water, electricity and toilet facilities (Table 1). The comparison group was more likely to reside in peri‐urban areas and less likely to own farmland and to be married. Most mothers had attended high school and were unemployed (∼70%).

**Table 1:** Baseline characteristics of households, mothers and children with Mullen Scales of Early Learning (MSEL) assessments at 9 or 18 months

	Comparison *n* (%)	Intervention *n* (%)	
	*n* = 181	*n* = 193	*p*‐value^*^
**Sex**			
Female	86 (47.5)	95 (49.2)	0.83
Male	95 (52.5)	98 (50.8)	
**Number of other children**			
0	33 (18.2)	29 (15.0)	0.86
1	57 (31.5)	60 (31.1)	
2	50 (27.6)	58 (30.1)	
3+	40 (22.1)	41 (21.2)	
Missing	1 (0.6)	5 (2.6)	
**Mother's education**			
Never attended	16 (8.80	21 (10.9)	0.14
Grades 1–2	4 (2.2)	1 (0.5)	
Standard 1–5	46 (25.4)	57 (29.5)	
Form 1–4	74 (40.9)	80 (40.5)	
Form 5	30 (16.6)	23 (11.9)	
University	8 (4.4)	2 (1.0)	
Missing	3 (1.7)	9 (4.7)	
**Marital status**			
Currently married	82 (45.3)	100 (51.8)	<0.001
Never married	49 (27.1)	64 (33.2)	
Other^a^	49 (27.1)	18 (9.3)	
Missing	1 (0.6)	11 (5.7)	
**Employment status**			
Unemployed	122 (67.4)	135 (70)	0.4
Employed	58 (32)	53 (27.5)	
Missing	1 (0.6)	5 (2.6)	
**Timing of ART initiation**			0.44
Before pregnancy	94 (51.9)	110 (57.3)	
During pregnancy	81 (44.8)	74 (38.5)	
After pregnancy	2 (1.1)	5 (2.6)	
Missing	4 (2.2)	4 (1.6)	
^*^ **SES quartile**			
Most poor	42 (23.2)	54 (28)	0.25
2	40 (22.1)	52 (26.9)	
3	65 (35.9)	53 (27.5)	
Least poor	31 (17.1)	29 (15)	
Missing	3 (1.7)	5 (2.6)	
**Type of residence**			
Rural	94 (51.9)	147 (71.2)	<0.001
Peri‐urban	86 (47.5)	41 (21.2)	
Missing	1 (0.6)	5 (2.6)	
**Electricity in household**			
No	74 (40.9)	96 (49.7)	0.069
Yes	104 (57.5)	92 (47.7)	
Missing	3 (1.7)	5 (2.6)	
**Clean/running water in household**			
No	85 (47)	104 (53.9)	0.133
Yes	94 (51.9)	84 (43.5)	
Missing	2 (1.1)	5 (2.6)	
**Toilet type**			
Pit latrine/none	166 (91.7)	183 (94.8)	0.082
Flush/pour	12 (6.6)	5 (2.6)	
Missing	3 (1.7)	5 (2.6)	
**Maternal depression status at baseline**			
EPDS<13	98 (54.1)	127 (65.8)	0.021
EPDS≥13	83 (45.9)	66 (34.2)	

^a^ Indicates divorced or cohabiting.

* SES scores: principal components analysis was used to develop a composite score of socio‐economic status based on reported household assets. Abbreviations: ART, antiretroviral therapy; EPDS, Edinburgh Postnatal Depression Scale; SES, socio‐economic status.

### 3.1 Neurodevelopment

Mean intervention arm scores were higher than comparison arm scores at 9 months in receptive language (55.7 [54.6, 56.8] vs. 53.7 [52.6, 54.8], *p*‐value 0.02); and at 18 months in expressive language (42.5 [41.6, 43.5] vs. 40.8 [39.8, 41.7], *p* = 0.01) and composite MSEL (85.4 [83.7, 87.2] vs. 82.7 [81.0, 84.4] *p* = 0.03) (Table 2).

**Table 2:** Estimated mean difference in Mullen Scales of Early Learning (MSEL) T‐scores (95% CIs and *p*‐values) from the mixed model adjusting for site clustering at 9 and 18 months

		Mean differences	95% CI lower limit	95% CI upper limit	*p*‐value
Visual reception	9 Months	−0.04	−2.07	1.99	0.97
	18 Months	1.85	−0.10	3.79	0.06
Fine motor	9 Months	−0.12	−2.07	1.84	0.91
	18 Months	1.32	−1.06	3.70	0.28
Receptive language	9 Months	1.99	0.39	3.58	0.02
	18 Months	0.99	−0.59	2.56	0.22
Expressive language	9 Months	1.48	−0.18	3.14	0.08
	18 Months	1.78	0.44	3.11	0.01
Gross motor	9 Months	0.19	−1.74	2.12	0.81
	18 Months	0.08	−1.79	1.95	0.78
Composite MSEL	9 Months	1.76	−1.26	4.78	0.25
	18 Months	2.71	0.29	5.14	0.03

Abbreviations: CI, confidence interval; MSEL, Mullen Scales of Early Learning.

For each domain, except gross motor, there was a significant decline in standard scores from 9 to 18 months (Table S2). Mean declines ranged from 9.4 points in fine motor to 25.1 points in expressive language (*p*‐values < 0.001), and 31 points in composite MSEL. Across all domains, the time‐by‐arm interaction terms were statistically non‐significant. No socio‐demographic characteristics significantly confounded the association between study arm and MSEL outcomes (Table S4).

### 3.2 Maternal depression

Comparison arm mothers had a higher prevalence of depression at baseline (83/181 [45.9%] vs. 66/193 [34.2%] *p* < 0.021) (see Table 1); reduced to 16.3% and 13.5% at 9 months, and 13.0% and 18.6% at 18 months, respectively. Random effects regression confirmed significant reduction in the comparison arm (prevalence ratio [PR] 0.35 [0.24, 0.49]) at 9 months, with no difference between study arms (time‐by‐arm interaction PR 1.14 [95% CI 0.68, 1.89]). At 18 months, the comparison arm reduction was significant (0.28 [0.18, 0.42]) and the intervention arm reduction attenuated, compared to the comparison arm (time‐by‐arm interaction PR 1.86 [1.06, 3.27]). Maternal baseline depression was not significantly associated with composite MSEL score across 9 and 18 months (mean effect 0.77 [−1.02, 2.55]).

### 3.3 Growth

The prevalence of stunting (Table S3) increased significantly to over 35% from 9 to 18 months across arms. There were no intervention effects on growth.

### 3.4 Caregiving activities

Median HOME Inventory scores were relatively high with no significant differences between the intervention and comparison arms (15 [13, 16], 16 [14, 16], respectively) and no associations with the composite MSEL score.

Among the six caregiving activities in the FCIs (Table 3), intervention‐arm mothers were more likely to report book‐sharing, story‐telling and possessing children's books than comparison‐arm mothers.

**Table 3:** Family Care Indicators

	Comparison		Intervention		
	*n*	Proportion	*n*	Proportion	Chi‐squared test *p*‐value
Shared books	164	0.41	180	0.63	<0.001
Told stories	164	0.54	180	0.66	0.03
Sang songs	164	0.91	180	0.93	0.65
Took the child out	164	0.66	180	0.58	0.1
Played with child	164	0.96	180	0.99	0.02
Named, counted with child	164	0.57	180	0.59	0.68

*Note*: Proportion of caregivers reporting activities with child, in the past 3 days, at 18 months.

### 3.5 Caregiving activities and mediation analysis

Mediation GSEM models were comprised of hypothesised direct effects: *study arm→MSEL scores* plus indirect effect: *study arm→book‐sharing→MSEL scores*. The indirect paths (mediation) were associated with increased scores across all MSEL domains (Table 4), *p*‐values ≤ 0.024. These indirect effects made up at least 45% of the total (direct + indirect) effects (Table S5).

**Table 4:** Mediating effect of book reading on relation between study arm and Mullen Scales of Early Learning (MSEL) scores, using generalised structural equation model

Visual reception		Coefficient	*p*‐value	95% CI (lower limit)	95% CI (upper limit)
	Direct effect	1.928	0.024	0.257	3.599
	Indirect effect	2.499	0.001	1.044	3.955
	Total effect	4.427	0.001	1.804	7.050
**Fine motor**					
	Direct effect	0.128	0.903	−1.917	2.172
	Indirect effect	3.242	0.001	1.331	5.153
	Total effect	3.370	0.016	0.629	6.110
**Receptive language**					
	Direct effect	0.397	0.621	−1.176	1.970
	Indirect effect	2.758	0.002	1.009	4.506
	Total effect	3.155	0.008	0.830	5.480
**Expressive language**					
	Direct effect	1.655	0.004	0.513	2.796
	Indirect effect	1.408	0.024	0.187	2.629
	Total effect	3.063	<0.001	1.642	4.484
**MSEL composite**					
	Direct effect	1.919	0.092	−0.313	4.151
	Indirect effect	4.583	<0.001	2.286	6.881
	Total effect	6.502	<0.001	3.172	9.833

Abbreviations: CI, confidence interval; MSEL, Mullen Scales of Early Learning.

### 3.6 Intervention contamination sensitivity analysis

Twenty‐four comparison arm mothers reported m2m/ECD contact. Excluding these mothers in a sensitivity analysis did not change the results (composite 18‐month MSEL scores: 85.5 [83.9, 87.2 vs. 82.5 [80.7, 84.3], *p* = 0.015).

## 4 DISCUSSION

This evaluation of a nurturing care intervention integrated into public antenatal clinics by Mentor Mothers and directed towards mothers of CHEU had three major findings. First, the intervention had significant effects on children's receptive language at 9 months and expressive language and composite MSEL scores at 18 months. Second, maternal depressive symptoms in both arms declined over the 18‐month study, with no significant intervention impact. Third, interactive caregiving activities, specifically book‐sharing, mediated the effects of the intervention on children's neurodevelopment.

Effects on children's language scores were significant, albeit modest. The entire distribution shifted with fewer children displaying language skills at the lower end of the distribution and more children at the upper end. This pattern is consistent with a meta‐analysis finding that parent‐implemented language interventions significantly improve both expressive and receptive language [35]. During infancy and toddlerhood, language skills are developing rapidly and sensitive to intervention [36]. Disparities in language development at 18 months widen over time and can impact negatively on children's behaviour and academic performance [37]. Thus, strategies to promote early language development can have long‐lasting benefits.

The language finding is encouraging evidence for future interventions based on nurturing care. The concept of nurturing care is based on evidence that children need nurturant and responsive relationships in a stable family environment, supported by communities and health and educational services [18]. Activities selected to promote nurturing care may vary to reflect cultural contexts and families’ strengths and challenges. Improvements in the intervention arm may reflect the sensitivity and training of the Mentor Mothers and appropriate cultural adaptions [38].

The relative decline in standard neurodevelopmental scores indicates a slower gain in skills between 9 and 18 months than expected. However, this finding is not uncommon in low‐resource settings [39]. Expectations for children's development increase with age and may reflect cultural variations. Slower gains in play and learning as children age are often reflected in a relative decline in children's standardised, age‐adjusted scores [40]. Interventions should be consistent with cultural variations while ensuring that children, regardless of HIV exposure, have access to opportunities that advance interactive play and learning.

Baseline maternal depressive symptoms were high among both arms, consistent with findings among other groups of pregnant women in Mentor Mother programmes in eSwatini [41]. Approximately half the sample (54.5%) initiated ART prior to pregnancy, suggesting that the remaining may have been newly diagnosed with HIV. In addition to concerns about their own HIV status, women may have been apprehensive about initiating ART and the health of their unborn infants [42]. Without data on the timing of the HIV diagnosis and possible co‐morbidities, the elevated prevalence of baseline depressive symptoms in the intervention group may suggest other unmeasured confounders. There was no association between baseline maternal depressive symptoms and MSEL scores at 9 or 18 months. Depressive symptoms in both arms declined over the 18‐month study, as expected [43], and may reflect women's adaptation to their infant's negative HIV status and their ability to parent their child. This finding is supported by relatively high scores in observed mother‐child interactions across both arms at 12–15 months. Strategies to mitigate maternal depressive symptoms through community health workers in low‐resource settings have been effective [44]; additional research is necessary to scale interventions to address HIV‐associated maternal depression.

Children's growth did not differ across arms. At 18 months, the prevalence of stunting increased to approximately 35%. This finding matches a recent UNICEF report of 35% stunting prevalence among children aged 18–23 months in eSwatini, regardless of HIV exposure, potentially linked to a severe 2015/16 drought that increased food insecurity [37]. Our intervention provided nutrition guidance and did not address the factors leading to the increase in stunting across both groups.

The finding that interactive caregiving activities, specifically book‐sharing, mediated the effects of the intervention on children's neurodevelopment provides suggestive evidence on the mechanisms underlying the intervention. Language skills are influenced by children's context, including verbal exchanges, [36] suggesting that caregiver‐child interactions and children's language skills responded to the intervention. Global studies, including southern Africa, have shown that early caregiving interactions, including book‐sharing, have beneficial effects on children's language and emerging literacy [16, 17, 18]. These findings suggest that conversational turn‐taking in interactive play drives advances in expressive language and visual reception, working through neural pathways central to language and learning [45]. The m2m intervention incorporated verbal interactions into daily activities, including feeding and bathing. Our findings suggest that book‐sharing and story‐telling increased in the intervention group and may be adaptable strategies that increase interactive caregiving interactions.

The findings should be interpreted recognizing several limitations. The clinics were not randomised to study arms. Intervention and comparison clinics were matched on multiple characteristics, analyses adjusted for identified socio‐demographic differences in location and marital status, and baseline maternal depressive symptoms were unlinked to children's neurodevelopmental scores. However, there may have been unmeasured confounding. Limited testing of children at older ages could have led to misclassification of their HIV status, though this should not have differed by arm and would be less likely with mothers receiving ART. Although the MSEL have been used among CHEU in other African countries, they were not standardised for eSwatini [4, 21]. In addition, we do not have an unexposed group of children for comparison. Finally, findings related to caregiving activities, including book‐sharing, were based on caregiver‐report and may have reflected respondent bias. Future studies should observe caregiving activities over time.

## 5 CONCLUSIONS

While the implementation of effective PVT strategies has decreased the number of infants living with HIV, the number of CHEU will continue to increase. The need to optimise the health of CHEU is urgent in countries such as eSwatini with large numbers of women living with HIV. CHEU are potentially at risk for poor neurodevelopment due to both prenatal exposure to HIV, and conditions associated with HIV‐exposed households [12]. CHEU may benefit from comprehensive interventions that address stable maternal functioning, promoting children's health and nutrition, and providing opportunities to learn and participate in responsive, emotionally supportive and developmentally enriching interactive caregiving activities [18, 46, 47]. The significant, albeit modest, differences in book‐sharing and story‐telling suggest that mothers incorporated book‐sharing into parent‐child play and learning activities associated with advances in children's neurodevelopment. Future randomised studies with focused interventions and longitudinal designs examining the impact of interventions on CHEU neurodevelopment are needed [48].

The m2m partnership is consistent with principles of implementation science where collaboration with a local team to design and implement and intervention promotes cultural sensitivity and ensures impact, and sustainability [49]. Investing in young children's development has benefits to CHEU and the larger society [18]. This study showed that an intervention based on nurturing care and integrated into PVT clinics, communities and homes in a resource‐limited country improved neurodevelopmental scores among CHEU. Our findings support recommendations from WHO and UNICEF that children worldwide, particularly in adverse conditions, receive interventions based on nurturing care [14]. Since our study was conducted, the number of CHEU needing nurturing care in eSwatini remains high. The Ministry of Health recognises the need to strengthen support programmes for CHEU. The country is a signatory to the Global Health Alliance and implementing a nurturing care framework for CHEU is a priority. Improving children's neurodevelopment can reduce disparities by building strong foundations for academic and economic capabilities.

## COMPETING INTERESTS

The authors declare that they have no competing interests.

## AUTHORS’ CONTRIBUTIONS

AR co‐conceived the study, served as a co‐principal investigator, oversaw all aspects of the study, interpreted the data, wrote pieces of the article and provided critical comments to the article. XD served as a co‐principal investigator from the Ministry of Health in eSwatini, oversaw aspects of the study and provided critical comments to the article. BASN conducted the data analysis, interpreted the data, wrote pieces of the article and provided critical comments to the article.

NS contributed to the study design; coordinated the data collection, data management and preparation of the final report; interpreted the data; wrote pieces of the article; and provided critical comments to the article. DK contributed to the study design, supervised the evaluators and child development specialists, and provided critical comments to the article. FB, ES and KS contributed to the study design, developed the m2m intervention, provided technical oversight of the m2m team in eSwatini that supervised the Mentor Mothers and provided critical comments to the article. FM and NG contributed to the study design, collected the neurodevelopmental data and provided critical comments to the article. LS contributed to the study design, contributed to data management and provided critical comments to the article. MMB served as a co‐principal investigator, contributed to the study design, trained the child development specialists, interpreted the data, wrote pieces of the article, provided critical comments and revisions to the article, and coordinated the preparation of the article. All authors provided critical comments on drafts, approved the final manuscript as submitted and agreed to be accountable for all aspects of the work.

## FUNDING

This study was funded by the United States Agency for International Development with PEPFAR funding [Cooperative Agreement #AID‐OAA‐A‐12‐00058] to the Johns Hopkins Center for Communication Programs.

## DISCLAIMER

The information provided in this article is not official U.S. Government Information and does not necessarily represent the views or positions of USAID, the United States Government or the Johns Hopkins University.

## Supporting information


**Table S1** Intervention content
**Table S2** Estimated mean MSEL changes (95% CIs) from the linear mixed model over 9 and 18 months, adjusting for site and child random effects, and arm by timepoint interaction terms
**Table S3** Number and percentage of children with z‐scores less than −2 on anthropometry, based on WHO standards
**Table S4** Predictors of composite MSEL score from the linear mixed model
**Table S5** Mediation effects of family care indicators on the relation between study arm and the composite MSEL score

## Data Availability

All the data files can be found in the data catalogue of the University of Maryland at Baltimore.
