# Supplementary material for: A trial of nurturing care among children who are HIV‐exposed and uninfected in eSwatini
Source: J Int AIDS Soc. 2023 Nov 1;26(Suppl 4):e26158. doi: 10.1002/jia2.26158 (PMC10618895; doi:10.1002/jia2.26158)
Supplement: Supplementary file 1 — Table S1 Intervention content Table S2 Estimated mean MSEL changes (95% CIs) from the linear mixed model over 9 and 18 months, adjusting for site and child random effects, and arm by timepoint interaction terms Table S3 Number and percentage of children with z‐scores less than −2 on anthropometry, based on WHO standards Table S4 Predictors of composite MSEL score from the linear mixed model Table S5 Mediation effects of family care indicators on the relation between study arm and the composite MSEL score [file JIA2-26-e26158-s001.docx]

| **A quasi-experimental trial of nurturing care**  **among children HIV-exposed and uninfected**  **Appendix** | |
| --- | --- |
| Supplemental Table 1 | Intervention Content |
| Supplemental Table 2 | Estimated mean MSEL changes (95% CIs) from the linear mixed model over 9- and 18-months, adjusting for site and child random effects, and arm by timepoint interaction terms |
| Supplemental Table 3 | Number and percentage of children with z-scores less than -2 on anthropometry, based on WHO standards |
| Supplemental Table 4 | Predictors of MSEL composite score from the linear mixed model |
| Supplemental Table 5 | Mediation effects of family care indicators on the relation between study arm and the composite MSEL score |

| Supplemental Table 1: Intervention content | | |
| --- | --- | --- |
| Essential Services | Focus | |
|  | Parent/Caregiver Level | Child Level |
| **Primary Level Maternal & Child Health** | - Referrals to ANC, PMTCT, HIV testing, ART - Understanding child development/ risks *in utero* - Mental health, substance abuse - Recognizing Common childhood illnesses | - Childhood illness (IMCI) - Referrals for Immunization, HIV, TB malaria services - Identification of developmental delay/ disability - Bathing/hand washing |
| **Nutrition** | - Breastfeeding & complementary feeding - Nutrition - Food security (referral for food/ nutritional support) | - Growth monitoring - Referral for micronutrient supplementation, deworming - Food security (referral for food/ nutritional support) |
| **Social Services** | - Referral for grants (where applicable) - Gender-based violence & trauma counselling | - Referral for birth registration - Referral to child protection services |
| **Stimulation for Early Learning** | - Understanding developmental milestones - Age-appropriate stimulation - Play/ stories/ songs/ dance/ talking/ culture - Access to resources (book/ toy libraries, etc.) - Toy-making | - Cognitive development - Socio-emotional development - Language development - Motor development - Visual-motor perceptual development - Exploration/ experimentation - Play groups |
| **Support for Primary Caregivers** | - HIV disclosure/ overcoming stigma - Coping skills - Nurturing parenting - Positive discipline - Involvement of fathers & household members - Home safety |  |

| Supplemental Table 2: Estimated mean MSEL changes (95% CIs) from the linear mixed model over 9- and 18-months, adjusting for site and child random effects, and arm by timepoint interaction terms | | | | |
| --- | --- | --- | --- | --- |
|  | Unadjusted | | | |
| Visual Reception | Mean Change | 95% CI low limit | 95% CI upper limit | P-value |
| Arm (intervention vs comparison) | 0.09 | -1.74 | 1.93 | 0.92 |
| Timepoint (18 months vs 9 months) | -16.93 | -18.53 | -15.34 | <0.001 |
| Arm by timepoint interaction | 1.72 | -0.50 | 3.94 | 0.13 |
| **Fine Motor** |  |  |  |  |
| Arm (intervention vs comparison) | -0.09 | -2.11 | 1.93 | 0.93 |
| Timepoint (18 months vs 9 months) | -9.37 | -11.09 | -7.65 | <0.001 |
| Arm by timepoint interaction | 1.31 | -1.08 | 3.71 | 0.28 |
| **Receptive Language** |  |  |  |  |
| Arm (intervention vs comparison) | 2.03 | 0.34 | 3.72 | 0.02 |
| Timepoint (18 months vs 9 months) | -12.43 | -13.77 | -11.09 | <0.001 |
| Arm by timepoint interaction | -1.07 | -2.93 | 0.80 | 0.26 |
| **Expressive Language** |  |  |  |  |
| Arm (intervention vs comparison) | 1.57 | -0.05 | 3.19 | 0.06 |
| Timepoint (18 months vs 9 months) | -25.09 | -26.29 | -23.88 | <0.001 |
| Arm by timepoint interaction | 0.16 | -1.52 | 1.83 | 0.86 |
| **Gross Motor** |  |  |  |  |
| Arm (intervention vs comparison) | 0.28 | -1.57 | 2.12 | 0.77 |
| Timepoint (18 months vs 9 months) | -1.23 | -2.54 | 0.09 | 0.07 |
| Arm by timepoint interaction | -0.28 | -2.10 | 1.55 | 0.77 |
| **Composite MSEL** |  |  |  |  |
| Arm (intervention vs comparison) | 1.92 | -0.97 | 4.81 | 0.19 |
| Timepoint (18 months vs 9 months) | -31.13 | -33.16 | -29.10 | <0.001 |
| Arm by timepoint interaction | 0.71 | -2.12 | 3.53 | 0.62 |

| Supplemental Table 3: Number and percentage of children with z-scores less than −2 on anthropometry, based on WHO standards | | | | | | | | |
| --- | --- | --- | --- | --- | --- | --- | --- | --- |
|  | 9 months | | | | 18 months | | | |
|  | Comparison (N=180) | | Intervention | | Comparison | | Intervention | |
|  |  |  | (N=188*) | | (N=166**) | | (N=180**) | |
|  | n | % | N | % | n | % | n | % |
| Weight-for-age | 15 | 8.5 | 18 | 9.6 | 9 | 5.6 | 15 | 8.33 |
| Length-for-age | 38 | 21.1 | 38 | 20.3 | 58 | 35.8 | 65 | 36.7 |
| Weight-for-length | 6 | 3.3 | 4 | 2.1 | 2 | 1.2 | 2 | 1.1 |
| MUAC-for-age | 5 | 2.8 | 4 | 2.1 | 0 | 0 | 0 | 0 |
| **^*^** 1 missing value; **^**^** 4 missing values **^***^**3 missing values | | | | | | | | |

| Supplemental Table 4: Predictors of MSEL composite score from linear mixed model | | | | | |
| --- | --- | --- | --- | --- | --- |
|  | Mean change | | 95% CI  (Lower limit) | 95% CI  (Upper limit) | P-value |
| Intervention vs Comparison | | 1.79 | -1.35 | 4.93 | 0.26 |
| 18 months vs 9 months | | -30.54 | -32.63 | -28.45 | <0.001 |
| Arm by timepoint interaction | | 0.55 | -2.36 | 3.46 | 0.71 |
| Male vs female | | -1.26 | -3.17 | 0.64 | 0.19 |
| **Mother's education (versus none)** | |  |  |  |  |
| Grades 1-2 | | -2.26 | -10.58 | 6.05 | 0.59 |
| Standard 1-5 | | -2.53 | -5.99 | 0.92 | 0.15 |
| Form 1-4 | | -1.72 | -5.12 | 1.67 | 0.32 |
| Form 5 | | -1.92 | -5.96 | 2.12 | 0.35 |
| University | | -5.31 | -12.38 | 1.75 | 0.14 |
| **Marital status**  **(versus married)** | |  |  |  |  |
| never married vs married | | -0.49 | -2.80 | 1.81 | 0.68 |
| Other | | -1.95 | -4.62 | 0.72 | 0.15 |
| **Employed vs unemployed** | | -1.62 | -3.74 | 0.51 | 0.14 |
| **Household SES (versus most poor)** | |  |  |  |  |
| 2 | | 0.68 | -1.97 | 3.32 | 0.62 |
| 3 | | 1.04 | -1.54 | 3.61 | 0.43 |
| least poor | | 2.20 | -0.95 | 5.35 | 0.17 |
| periurban versus rural | | 1.72 | -1.41 | 4.86 | 0.28 |

| **Supplemental table 5: Mediating effect of family care indicators on relation between study arm and composite MSEL scores, results for the generalized structural equation models** | | | | | |
| --- | --- | --- | --- | --- | --- |
| Care indicator |  | Coefficient | P-value | 95%CI lower limit | 95%CI upper limit |
| Tell stories | Direct effect | 2.97 | 0.01 | 0.66 | 5.28 |
|  | Indirect effect | 0.14 | 0.83 | -1.14 | 1.42 |
|  | Total effect | 3.11 | 0.02 | 0.43 | 5.78 |
|  |  |  |  |  |  |
| Play with child | Direct effect | 2.80 | 0.02 | 0.50 | 5.09 |
|  | Indirect effect | 10.06 | 0.15 | -3.68 | 23.80 |
|  | Total effect | 12.85 | 0.08 | -1.33 | 27.03 |
|  |  |  |  |  |  |
| Sing songs | Direct effect | 2.97 | 0.01 | 0.64 | 5.30 |
|  | Indirect effect | 0.17 | 0.83 | -1.44 | 1.79 |
|  | Total effect | 3.14 | 0.03 | 0.30 | 5.99 |
|  |  |  |  |  |  |
| Take out | Direct effect | 3.04 | 0.01 | 0.67 | 5.42 |
|  | Indirect effect | -0.30 | 0.62 | -1.45 | 0.86 |
|  | Total effect | 2.75 | 0.02 | 0.50 | 4.99 |
|  |  |  |  |  |  |
| Name, count and draw | Direct effect | 2.83 | 0.02 | 0.55 | 5.12 |
|  | Indirect effect | 0.35 | 0.69 | -1.39 | 2.10 |
|  | Total effect | 3.19 | 0.03 | 0.31 | 6.06 |
